# Cold, Hot and Warm Applications in the Treatment of Diseases of the Eye

**Published:** 1884-01-20

**Authors:** J. Morrison Ray

**Affiliations:** House Surgeon to the Manhattan Eye and Ear Hospital


### COLD, HOT AND WARM APPLICATIONS IN THE TREATMENT OF DISEASES OF THE EYE.

By J. Morrison Ray, M.D.
**House Surgeon to the Manhattan Eye and Ear Hospital.**

When casually considered, these agents may seem of but little importance, not likely to do harm, and capable of little good But a more careful study will show that in the hands of those ignorant of their value or indications they may become most dangerous agents, and that when judiciously applied they rank among the most effective remedies in the treatment of many affections of the eyes. The exact indications for the use of these agents are not always plain, since it is often the case that in two patients suffering from the same disease, the remedy which would do good in the one might do harm in the other.

In acute catarrhal inflammations of the conjunctiva, frequent bathing of the eyes with cold water, or, in more severe cases, the application of iced cloths,* will often give prompt relief, and may effect a cure. As a rule, however, better results will be obtained if a mild astringent, such as alum or zinci sulph. (gr. ii–iv–℥j), or the solution recommended by Dr. Agnew, be used as supplementary to the cold applications.†

The lids being everted, the solution is sprayed, by means of Davidson's hand atomizer, on the conjunctiva. This may be done several times a day. Besides acting as an astringent, the solution thoroughly cleanses the conjunctival surface of all secretions.

In gonorrhœal ophthalmia or in ophthalmia neonatorum, where the inflammation runs high, the discharges being thick and copious, and the lids swollen and tense, the methodical application of ice cloths, day and night, with frequent cleansing of the conjunctiva by means of pure water, or preferably a saturated solution of boric acid, will often check destructive processes, and give the patient relief from the pain. If the disease has not run a severe course, or is on the decline, a milder form of the same treatment will suffice. If the inflammation does not readily yield to this treatment, astringents, or very mild caustics, are indicated.

In the conditions just cited the application of fomentations or poultices would be followed by the most serious consequences, permanent injury to or destruction of the cornea being a common result.

In the first stage of granular conjunctivitis, where there is much inflammation, iced cloths are useful, checking inflammation to some extent and allaying irritation. Later on, when this condition becomes chronic, with its characteristic hard trachoma granule, there being very little inflammatory action present, hot water is often beneficial, and may even be curative. By frequent applications a certain amount of irritation is aroused, accompanied by a swelling of the conjunctiva and a softening of the trachomatous masses, which tend to hasten their absorption. This can be so regulated as to keep up a continuous slight irritation, and thus often a rapid disappearance of the granulations, without even the use of caustics, which have a tendency to cause cicatricial contraction of the conjunctiva, followed by all the bad symptoms incident to this condition, trichiasis, entropion, etc.

In acute inflammations of the cornea, especially when due to

---

*Take a lump of ice the size of a brick and place on it bits of linen two inches square. When they get cold transfer to eye, and then again to ice when they become warm.

†Ac. tannici, sodæ biborat, each ten grains; glycerine, one dram; aq. camphoræ, ad. one ounce. Filter. To be used in connection with the cold application.

traumatism, iced cloths often give great relief by allaying the pain and limiting the inflammation. In phlyctenular keratitis accompanied by photophobia, which is often seen in badly nourished ehildren, the dropping of iced water on the exposed cornea has been recommended. I have recently seen a case which demonstrates the efficacy of this treatment to a marked degree. The child had been treated by other methods for some time without any apparent benefit. Under the use of iced water, dropped on the forcibly exposed cornea, the improvement was remarkable.

In necrosis of the cornea, occurring in strumous children, non-inflammatory in origin, and without any conjunctival irritation, our chief reliance is in the persistent use of fomentations made of chamomile flowers or poppy-heads.

In abscess of the cornea, accompanied by hypopyon, or the formation and deposit of pus in the anterior chamber, fomentations allay the pain and promote the absorption of the pus. In that form of ulcer of the cornea called serpiginous, occurring at its margin as an ulcerated band with irregular edges with a tendency to spread circumferentially (a condition often seen in old, feeble and debilitated subjects), and accompanied by considerable conjunctival irritation and a deep ciliary injection, with often excessive pain and photophobia, rapid improvement sometimes follows the application of heat, moist or dry. The latter is to be preferred if the former produces much swelling of the lids or edema of conjunctiva. The hot applications may be supplemented by a sol. of sulph. eserine (gr. $\frac{1}{4}$–gr. j–℥j), dropped in eye several times a day. This is often used with marked benefit. Careful attention must be given to hygienic surroundings. Exercise in open air several times a day should be enjoined, during which the eyes must be protected from bright light by means of colored glasses.

The above treatment, if carried out properly, has often succeeded in cases where incision of the base of the ulcer (Soemiesch's incision) has failed. In interstitial keratitis hot fomentations assiduously applied, their effect being carefully watched, together with frequent instillations of atropine, are sufficient to allay the pain and ciliary irritation. In suppuration of the cornea after cataract extraction, especially if it be in a weak and debilitated subject, heat in some form is always serviceable. There is generally considerable chemosis of the conjunctiva and swelling of lids, and in this condition dry heat would seem to be indicated. These eyes are, however, usually doomed, and iced cloths often tend to limit the inflammatory condition; but if this be once fully established, the hot water will promote the suppurative process, which ultimately terminates in phthisis bulbi.

In iritis, atropine to dilate the pupil, preventing adhesions and putting the eye at rest, and the frequent application of hot water to allay the pain and ciliary neuralgia make an excellent treatment.

Frequently a Turkish bath has a marked effect on these cases. I have often seen a pupil dilate under atropine just after a Turkish bath, which before would give no response to the drug, no matter how persistently applied. Traumatic iritis is the only form which will bear the application of cold, and in this the patients often prefer heat, especially if there is a suppurative process going on in the part.

In inflammation of the deeper tunics of the eye, if accompanied by much ciliary irritation or neuralgia, hot will be agreeable and often beneficial. In sympathetic inflammations hot poultices of flaxseed or bread and milk continuously applied for some time has been said to be followed by good effects. Ayers reports a case in which poultices were used almost continuously for four months and with marked improvement in the condition of the eye. In inflammation of the circumocular fibrous and cellular tissue, cold continuously applied for hours at a time will tend to diminish the heat and swelling of the part and relieve the pain. If it be desired to expedite the suppurative process, which often can not be prevented, hot water would be in order. It can be seen from what has been said that no strict rules or rigid laws can be laid down as to the use of these agents. Potent for good in one case, they may produce the opposite effect in another suffering from a similar condition. It would seem that the following would be indications for their use in general: In acute inflammations, followed by much elevation of temperature or swelling of the part, or in any condition where a lessening of the vascular action is required, cold in some form, dry or moist, intermittent or continuous, is indicated, and generally gives the required relief. Where an increase in the blood-supply of a part is desired, or when the vitality is threatened by a slow necrotic rather than an inflammatory process, heat in some one of its modes of application is clearly indicated.—*Louisville Med. News.*

